# A Comparative Analysis of Methods of Endmember Selection for Use in Subpixel Classification: A Convex Hull Approach

**DOI:** 10.1155/2022/3770871

**Published:** 2022-10-12

**Authors:** Vidhya Lakshmi Sivakumar, K. Ramkumar, K. Vidhya, B. Gobinathan, Yonas Wudineh Gietahun

**Affiliations:** ^1^Department of Civil Engineering, Saveetha School of Engineering, SIMATS, Chennai, India; ^2^Department of Computer Science and Engineering, SRM Institute of Science and Technology, Vadapalani, Chennai, India; ^3^Department of Electronics and Communication Engineering, Saveetha School of Engineering, SIMATS, Chennai, India; ^4^Jaya Sakthi Engineering College, Thiruninravur, Chennai, India; ^5^Department of Chemical Engineering, College of Biological and Chemical Engineering, Addis Ababa Science and Technology University, Addis Ababa, Ethiopia

## Abstract

Mixed pixels in aerial and satellite images are common, especially near the boundaries of two or more discrete classes; that is, they tend to occur at the transitional region between two classes. Ideally, to decipher the mixed pixel, a soft classification is performed compared to a hard- or a per-pixel classification. Soft or subpixel classification is carried out where the fractional cover of the LULC contained within a pixel is derived. Endmembers are extracted for three VNIR bands of ASTER data for two image datasets using three approaches, namely, principal component analysis (PCA), pixel purity index (PPI), and convex hull-Graham scan (CHGS). On comparing the DN values of the identified endmembers, it is observed that the CHGS method provides the most appropriate end members than the PCA-derived and PPI-derived end members. This is based on deriving the endmembers from two different image conditions. Convex hull implemented using the Graham scan algorithm delineates the pure pixel and pinpoints the exact number of endmembers. These accurate end members would result in accurate proportions of the land cover for better modeling of the terrain.

## 1. Introduction

Remote sensing image classification helps in the mapping of land use/land cover for the monitoring and understanding of various physiological and climatological changes happening on the Earth's surface. The result of the classification is generally a thematic map that aids in several remote sensing applications. Scientists working on image classification have been successful in obtaining the output for the investigation. However, the accuracy of the classification depends on several factors since image classification is a complex process. The complexity arises due to the changes and corrections applied to atmospheric and radiometric calibration, LULC of the region, and the available techniques (Lu and Weng). With the increase in resolution of the sensors in recent days, accuracy increases with increased resolution as observed in. However, there is still a lack of availability of high-resolution data, and other techniques such as image fusion are sought after. An alternative approach to deal with this is to resort to mixed pixel classification or soft classification. This is known as subpixel classification. Subpixel classification provides the user with the fractional cover of LULC within a pixel. Ideally, subpixel classification requires pure pixels as input to the algorithm. Unless there is sufficient ground truth information available for a scene, the problem of mixed pixel and therefore, the identification of a pure pixel becomes difficult. This study discusses a few methods that are conventionally used for the extraction of pure pixels of land cover components, their merits, and demerits and also introduces a new method for the selection of the end members using the principles of convex geometry.

Endmembers are also known as pure pixels that consist of a single LULC class. An LMM is modeled as a linear function of spectral reflectance of endmembers to derive the fractional area of land cover classes. The quality of the fraction images derived from spectral unmixing depends on a sufficient number and the proper selection of end members. It is also believed that an exact number of end members is required to account for the spectral variability of the scene (Sabol et al.). Spectral unmixing requires accurate, well-characterized endmembers (Elmore et al.; Milton; Tompkins et al.). If the end members are not obtained properly, i.e., improper selection of end members leads to erroneous computation of fractions of LULC classes present in an image. Therefore, the concept of an appropriate spectral endmember is fundamental to the technique of spectral unmixing. The identification and description of the physical and spectral properties of endmembers are thus of great importance in spectral unmixing (Milton; Tompkins et al.) [1–20].

## 2. Selection of Endmembers

There are several algorithms for the selection of endmembers to be used in spectral unmixing. A few of them are inputs from field-based or lab-based spectra, transformation-based inputs, MESMA, n-dimensional visualization tools, etc. Most such techniques derive the endmembers from the image. The advantage of using image endmembers in unmixing is that image scene variability can be taken into account when image endmembers are utilized to produce fraction images. Therefore, it is possible to model the image in a better fashion. In this study, endmembers are derived from three techniques, namely, pixel purity index (PPI), principal component analysis (PCA), and a new approach. These three methods were chosen because their performance varies depending on the preprocessing techniques they employ, such as initial conditions and dimensionality reduction transforms. This study proposes a novel endmember extraction technique based on convex hull property ensembling the scatter plot of the DN values from two bands. A comparison of these three techniques in terms of the digital numbers of the image endmembers is carried out and the results are discussed.

### 2.1. Principal Component Analysis (PCA) Approach

Multispectral image bands are often well correlated. This can be attributed to several factors such as material spectral correlation, topography, and sensor band overlap (Schowengerdt). Therefore, analysis of such correlated bands is difficult due to redundancy arising from the above-mentioned factors. Therefore, it is required to reduce the dimensionality of the large and multiple band dataset using transformation (Jollife and Cadima). Principal component analysis is one such technique that is widely used. A feature space transformation for removing this redundancy is the principal component transformation (PCT). The result of the transformation is a set of principal component images, which determine the intrinsic dimensionality of the data. The principal components are based on the eigenvectors of the covariance and the correlation matrix. The eigenvector associated with the largest eigenvalue has the same direction as the first principal component. The principal components are obtained in the order of decreasing variance. This means that the first PC accounts for most of the variance, the second for lesser variance, and so on (Lillesand et al.). The decorrelation and high measures of statistical significance provided by the first few principal component axes are no guarantees of having the best subset of features. Nadler suggests that PCA finds feature combinations that model the variance of a data set, but these may not be the same features that separate the classes, i.e., the PCA components that model the largest contributions to the data set variance may work poorly for pattern recognition. The principal component analysis can be used for the following applications:

- Effective classification of land use with multiband data.
- Color representation or visual interpretation with multiband data.
- Change detection with multitemporal data.

It is well known that PC1 and PC2 are two bands with very low correlation. The spatial distribution of the pixels in a two-dimensional feature space can be seen in the scatter plots created by charting the various principal components. The plot's form provides an estimate of the number of endmembers (Bryant and Gilvear; Schowengerdt; Shanmugam and Barnsley). This indicates that the number of endmembers correlates with the number of vertices in the data cloud of the scatter plot of PC1 vs. PC2 (Ann Bateson et al.; Bateson and Curtiss). As a result, a triangular plot is likely to have three endmembers, while a rectangular plot is likely to have four endmembers. The vertices or outermost corners of the data cloud are where the plot's purest pixels are found. The scatter plot's additional pixels stand in for the scene's mixed pixels. Therefore, the vertices of the polygon in the plot are the endmembers that may be chosen from the scatter plot of PC1 against PC2. When compared to other methods, PCA takes less processing time and maximizes the amount of data variance and signal-to-noise ratio in the visual scene (Dópido et al.; Karamizadeh et al.).

### 2.2. Pixel Purity Index (PPI) Approach

Pixel purity index (PPI) is another technique for the delineation of the pure pixel, which involves repeatedly projecting n-dimensional scatter plots onto a random unit vector (Boardman et al.). The highest and lowest reflectance pixels of the projection are scored including any other pixels located within a specified standard deviation range (i.e., the designated threshold value). Pixels with extreme values are scored most often and represent the corners (i.e., vertices) of the multidimensional cloud of data points (Boardman et al.). By understanding the vertices in the plot, it is possible to understand the spectral mixes in those LULC features. PPI, like PCA, allows spatial data reduction. Pixels in the image with the “most pure” spectral signatures are identified. Such pixels are taken as endmember pixels representing the mixed pixels. The PPI is carried out on MNF (maximum noise transform) image data suggested by Green et al. This is because MNF determines the intrinsic dimensionality of the image data excluding noise and decreases the computation burden of the algorithm in the further processes to be carried out (Boardman et al.).

The result of the application of PPI is a PPI score image with bright and dark pixels, each pixel having a distinct score. The PPI score indicates the purity of the pixel. A pixel with a higher PPI score indicates higher purity and hence, appears brighter in the image. Pixel with the lower score is less “spectrally pure” and appears darker in the PPI image. Pixels with higher PPI scores are usually taken as endmembers for use in spectral unmixing (Boardman et al.). It must be noted both PPIs require the number of endmembers for the image scene to be specified in advance (Tao et al.). The simplest method of implementation of endmember extraction is PPI (Heylen and Scheunders). However, the use of PPI is limited and is accessible in the Envi software package (Chang and Plaza).

### 2.3. Limitations of PCA and PPI Approaches in Selecting Endmembers

Though there are several advantages of PCA and PPI techniques for the selection of the endmembers, some of the drawbacks of the same have been identified in this study.

PCA technique fundamentally finds the intrinsic dimensionality of the image data and the scatter plot containing the principal components gives the location and number of the endmembers. The derivation of the endmembers from such a plot is basically through manual/interactive means by the analyst. This interactive method involves two kinds of errors:

- The vertices may contain a group of pixels, which may not correspond to the pure pixel. In case of such a selection of more than a pixel, mean statistics of the pixels representing a land cover class are taken as an input for unmixing.
- The selected pixel may not correspond to the purest pixel in the plots of principal components.

Thus, there is a possibility of the pure or wrong selection of pure pixels representing the land cover class, which is mainly attributed to manual error. This kind of manual error is common while using the PCA technique for the selection of endmembers. PCA performs a dimensionality reduction of second-order statistics, which means that endmembers are information of higher order with noise getting removed because of the increase in inherent intrinsic dimensionality of the data with spectral bands. Hence, PCA-based transforms result in endmembers that are not pure enough to be input in spectral unmixing.

PPI, on the other hand, gives the purest pixels with a high PPI score.

In reality, it is observed that pixels with a high PPI score do not represent the purest pixels. The case of mapping different vegetation species of a region is considered. A PPI image of such a region would only provide three to four pixels with a high PPI score neglecting the different species of vegetation for mapping the region. Pixels with a lower score of PPI may correspond to an endmember representing a species of vegetation. It may also happen in such a way that the pixels with higher scores correspond to a single species of vegetation. PPI may not produce as many or as finely separated endmembers as a user would desire. Therefore, PPI does not produce pure pixels to the expectation of the analyst and lacks in providing the exact number and type of endmembers. The PPI used in this study is derived from Envi 4.5, an image processing software. Envi is used in several applications successfully (Qi et al.). The PPI algorithm has been widely used in the remote sensing community due to its publicity and availability, provided by Research Systems' ENVI software package (Plaza and Chang).

Envi uses two parameters to determine the final set of endmembers for the PPI calculation: *k* (random vectors referred to as skewers) and *t* (cutoff value). It has been noted that the PPI data obtained from the Envi do not adhere to the iterative process, which can prevent the genuine endmembers from being displayed. Consequently, a fast iterative PPI was put into place (Chang and Plaza). Additionally, the original set of skewers used to implement PPI cannot be changed (Chaudhry et al.). However, in this work, the endmembers are derived using the PPI's ENVI version.

While the error involved in the PCA technique for the derivation of the endmembers is attributed to manual and interactive reasons, the error with the PPI technique is that of an algorithm/system-based error. Hence, a new method for the derivation of pure pixels from the scatter plots is required. In this study, an approach is suggested based on the original spectral reflectance of the land cover classes without any need for the transformation of the satellite image data. The algorithm and method of derivation of endmembers using the approach are discussed in the following sections [20–35]:

### 2.4. The Convex Hull Algorithm

Considering the lack of the above-said factors for the selection of endmembers, in this study, a novel approach, namely, the convex hull approach is proposed to exactly pinpoint the vertices of the scatter plot, i.e., the most accurate endmembers (Graham). Since endmembers tend to be pure for any component, they occur at the extreme corners of a scatter plot of image data (Schowengerdt; Shanmugam and Barnsley). Therefore, the concept of the convex hull is helpful in the delineation of pure pixels for use in spectral unmixing, which has been adopted.

#### 2.4.1. Principles of Convex Hull

The convex hull for a geometrical object or a set of geometrical objects is the minimal convex set containing the given objects. For planar objects, i.e., lying in a plane, an easy way to visualize the convex hull is to imagine a rubber band tightly stretched to encompass the given objects; when released, it will assume the shape of the required convex hull.

Computing the convex hull means that a nonambiguous and efficient representation of the required convex shape is constructed. The complexity of the corresponding algorithms is usually estimated in terms of *n*, the number of input points, and *h*, the number of points on the convex hull known as big O notation. Big O notation is a symbolism used in complexity theory, computer science, and mathematics to describe the asymptotic behavior of mathematical functions. The most common representation is the list of vertices of a set of points ordered along its boundary clockwise or counterclockwise.

There are many algorithms to construct a convex hull for a given set of points. Two of the most widely used methods are as follows:

1. Gift-wrapping algorithm
2. Graham scan algorithm

#### 2.4.2. Graham Scan Convex Hull (GSCH) Algorithm

Graham scan, who dealt with O (n log n) time complexity, introduced the Graham scan convex hull as a sophisticated approach. The Graham scan algorithm is used to generate a convex hull, and it does so quickly and reliably. The steps involved are as follows:

The gift-wrapping algorithm has an O (n, h) complexity, where *h* is the total number of points on the hull. Due to the fact that it requires less checking when there are fewer points on the hull, this algorithm performs well. When several (or all) points land on the hull, a problem occurs. If so, it becomes clear that the complexity is much worse than the Graham scan algorithm, rising to O (n, n) or O (n2). Endmembers are seen near the corners of a scatter plot of picture data because they are often pure for any component. In order to distinguish pure pixels for use in the widely used spectral unmixing method, the convex hull notion is useful.

**Figure 1:**
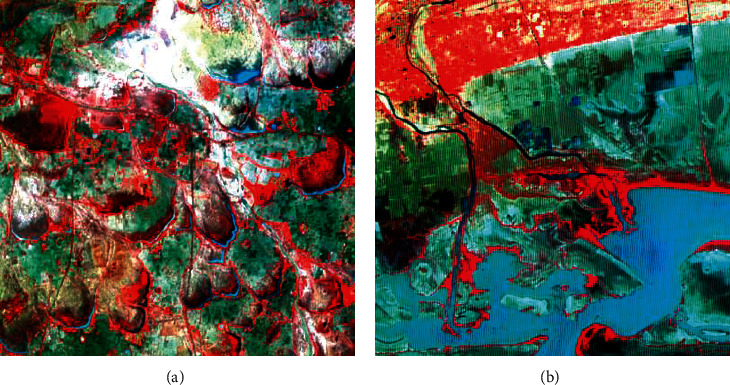
FCC of (a) study area 1—Manamelkudi and (b) study area 2—Vedaranyam (red = band 3, green = band 2, and blue = band 1).

To demonstrate the advantages of the convex hull algorithm over PCA and PPI approaches, images of ASTER VNIR and IRS 1C LISS-III sensors were used for this study (Figure 1). ASTER (Advanced Spaceborne Thermal Emission and Reflection Radiometer) is an imaging instrument that is flying onboard TERRA-1, a satellite launched in December 1999 as a part of NASA's Earth Observing System (EOS). ASTER has three spectral bands in the visible near-infrared (VNIR), six bands in the short wave (SWIR), and five bands in the thermal infrared (TIR) regions, with 15, 30, and 90 m spatial resolution. The ASTER data product is Level 1B data, which contains radiometrically calibrated and geometrically coregistered data for all the bands (Yamaguchi, Kahle, Tsu, Kawakami and Pniel). Three bands of ASTER visible near-infrared (VNIR) data were used in this study.

### 2.5. Study Regions and Dataset Used

Two regions were selected for this study located near (a) Manamelkudi and (b) Vedaranyam to demonstrate the principles of the convex hull. These regions were chosen since they exhibited good and diversified land use/cover patterns. In addition, the scatter plots of DN values using band 2 and band 3 as well as the principal component plot showed a four-limbed spatial distribution of the points. Therefore, the study area is apt for the demonstration of the concept of a convex hull and its use in the delineation of exact endmembers as an input to spectral unmixing.

ASTER (Advanced Spaceborne Thermal Emission and Reflection Radiometer), an imaging instrument that is flying onboard TERRA-1, consists of three spectral bands in the visible near-infrared (VNIR), six bands in the short wave (SWIR), and five bands in the thermal infrared (TIR) regions. While the VNIR has a 15 m spatial resolution, the SWIR and the TIR bands have a 30 m and 90 m spatial resolution, respectively. ASTER VNIR bands can provide stereoscopic images due to a backward-looking camera. ASTER image of the study area (Figure 1) in the VNIR region with a spatial resolution of 15 m was used for this study. The ASTER data product is Level 1B data, which contains radiometrically calibrated and geometrically coregistered data for all the bands (Yamaguchi, Kahle, A. BTsu, Kawakami, and Pniel). The fourth in the IRS series, IRS 1C consists of three sensors, namely, a panchromatic camera (PAN), linear imaging and self-scanning sensor (LISS-III), and wide field sensor (WiFS). LISS-III camera provides multispectral data in 4 bands used for this study (IRS 1C Handbook). The spatial resolution for visible (two bands) and near-infrared (one band) is 23.5 m with a ground swath of 141 km. The fourth band (SWIR band) has a spatial resolution of 70.5 m. Three bands with a resolution of 23.5 m were used for the demonstration of the principles of a convex hull. Three bands of ASTER-VNIR and IRS LISS-III data were used for the extraction of endmembers using various approaches to endmember extraction and the spectra thus obtained are compared.

### 2.6. Applying Different Approaches to Endmember Selection

#### 2.6.1. Derivation of Endmembers Using the PCA Approach

The PCA images were obtained by analyzing the Eigenvalues applying PCT and the variance of the first component for the two study regions were 86.91% and 76.29%, respectively. The PC scatter plot for the study area was obtained by plotting the first principal component against the second principal component, which exhibited a highly scattered distribution of the pixels, which is due to the inherent dimensionality of the image data. The endmembers for the area were chosen manually by marking corner pixel/pixels in the plot and linking it with the image. The spectra of the endmembers were noted down.

#### 2.6.2. Derivation of Endmembers Using the PPI Approach

PPI images for both study areas (1) and (2) were derived with the number of iterations being 1,00,000 when the PPI curve reached a stable straight line. The PPI curve is an indicator of the number of pixels being “spectrally pure” and the straight line indicates that the pure pixels have been recorded as extreme pixels. The PPI images were then linked with the FCC of the corresponding images to obtain the brighter pixels, and hence, the spectra of the pixels with higher PPI scores were noted down for each of the land cover components.

#### 2.6.3. Delineation of Endmembers from Convex Hull Algorithm

In this study, the exact number and type of pure pixels reflecting the number of land cover components were delineated and chosen using the Graham scan algorithm. In order to accomplish this, software was created utilizing the C and IDL programming languages that extract the endmembers from a set of input points. All of the pixels' DN (spectral reflectance) values for each band in the image scene serve as the input. All of the pixels' band 1 values, band 2 values, and so on are defined by the algorithm. The method then calculates a collection of points sitting on the convex hull based on the user-selected band for the convex hull's formation. Plotting the resulting collection of points yields a convex hull, which may be used to identify the pure pixels and use them as input for the spectral unmixing operation.

### 2.7. Results and Discussion

The endmember spectra for various land cover components from various approaches are listed in Tables 1 and 2. Tables 1 and 2 list that there is explicit variance in the DN of endmembers defined by different techniques. Considering the DN of vegetation for the three bands in Table 3, while PCA and convex hull method give spectral values typically akin to vegetation, PPI results in providing “not a true pixel” with spectral values not representative of a vegetation class. A value of 123 in the third band, namely, the NIR band, which is lesser than 124 in the first band (green band) is evidence of the provision of “not a true pixel” by the PPI technique.

#### 2.7.1. Endmembers from PCA Approach

**Figure 2:**
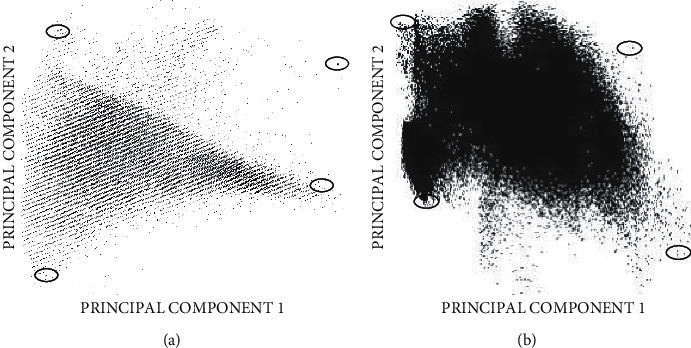
Scatter plot of PC1 against PC2 showing the locations of the endmembers for (a) study area 1 and (b) study area 2.

The PCA scatter plots for the study regions are shown in Figure 2. The plot shows a four-limbed distribution of the pixels for both the Manamelkudi and Vedaranyam study areas. This suggests that there are four endmembers in the image scene. Examining the plot reveals that there are many pixels concentrated around the vertices of the plot. Therefore, there is uncertainty in the selection of a pure and extreme pixel from such grouped pixels. The clustered pixels in Figure 2(a) make it challenging to distinguish the vegetation endmember from the lower right corner. Figure 2 shows that study area 2 has a worse situation (b). It is difficult to infer the endmembers since the pixels are densely crowded at all of the vertices. The vegetation endmember produced even from such clustered and dispersed pixels is not typical because the DN in the NIR band (102) is lower than that of the green band (109). If this fake endmember was used for spectral unmixing, the components of the land cover would be disproportionate.

#### 2.7.2. Endmembers from the PPI Approach

**Figure 3:**
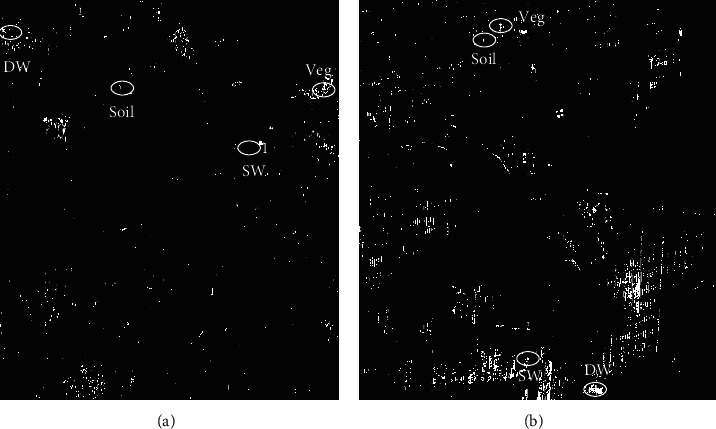
PPI images of (a) study area 1 and (b) study area 2 showing the location of the endmembers. DW, deep water; SW, shallow water; Veg, vegetation.

Figure 3 displays the PPI images for the research locations. Slices of the density levels of the PPI scores were used to choose the endmembers for four classes. High PPI-scoring pixels were used as endmembers. It is seen from Table 3 that the endmember spectrum for vegetation has a low DN value of NIR of 123 than the green band (124). This pixel was the only vegetation pixel with a PPI score as high as 11861, and therefore, this pixel was chosen as the endmember for vegetation. While PPI for soil and deep water was found to have a maximum value of 22964 and 24362, respectively, there was no pure pixel corresponding to shallow water. This suggests that the purity of the pixels provided by PPI needs to verify.

A similar observation for study area 2 for that of vegetation endmember as the spectra have a lesser DN value (101) in NIR than that of the green band (104) raising doubt about the purity of the pixel. It was also noted that while the pixels of soil and shallow water had a score of 25418 and 20005, the pixel delineated as the endmember for deep water had a very low score of 4523. This also provides strong evidence of the fewer pure pixels provided by the PPI approach.

#### 2.7.3. Endmembers from the Convex Hull Algorithm

**Figure 4:**
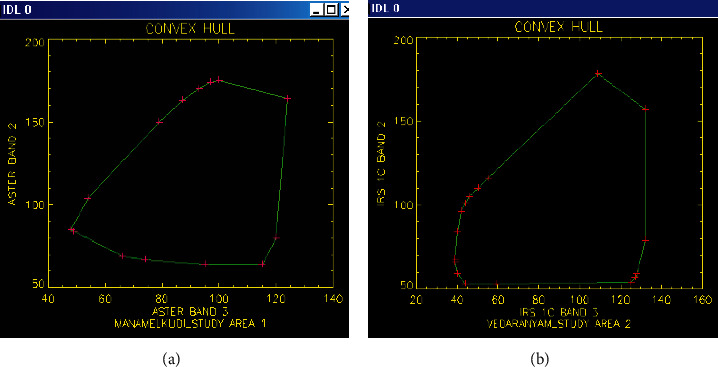
Plot showing the convex hull obtained using Graham scan algorithm for the (a) study area 1—Manamelkudi and (b) study area 2—Vedaranyam.

The endmember spectra for each class using a convex hull were delineated as listed below. The convex hulls for the study areas are shown in Figures 4(a) and 4(b). It was observed that all the points lying on the convex hull were not endmembers, but the pixels at the vertices of the polygon enclosed by the hull are endmembers. It can be seen from Figure 4(a) that there exist two pixels for water in the lower left corner of the hull. The DN of these two pixels was 123, 85, 48, and 125, 84, 49 (corresponding to b1, b2, and b3). It is logical to note that the DN of water is high in band 1, and therefore, the pixels with the DN 125, 84, and 49 were chosen as the endmember. A similar reason holds for vegetation pixels at the lower right corner of the hull. The two pixels have DN of 99, 64, 115 and 118, 80, and 120. Of these, the latter was identified as the endmember since it has a high value in the NIR band.

For study area 2, there are two pixels of vegetation in the proximity of the lower right corner of the convex hull. The DN of the two pixels was 97, 57, 127, 111, 59, and 128, respectively. The latter was taken as endmember since it has a high DN in the NIR band. Similarly, there are three pixels in the lower left corner of the hull, out of which the pixel with DN 40, 59, and 93 was chosen as water endmembers. Thus, it is possible to delineate the pure pixel for the land cover components with the use of a convex hull approach.

#### 2.7.4. Comparison of the Three Approaches

The vegetation spectra from Table 1 are correctly provided by the convex hull. Though PCA represents vegetation spectra, convex hull gives exact spectra of the same. The vegetation spectra derived from PPI are “not a true pixel.” Therefore, a convex hull provides accurate spectra for vegetation. The endmember spectrum of soil is the same as derived by the three techniques, whereas a slight variation is observed in the case of deep water. The spectrum of shallow water is identical for the three approaches. A similar reason quoted for vegetation endmember for Manamelkudi holds for vegetation spectra in the Vedaranyam. The spectrum of shallow water is the same for the three techniques. Considering the spectra given by the convex hull for shallow water, the PPI technique presents with a score of 18992 for the same pixel location. This provides strong evidence for the false pure pixel generated by the PPI. Considering the soil spectrum PCA and PPI provide a typical endmember, a convex hull produces an endmember for the soil with higher values in all the bands. There is no notable difference in the endmember spectra of shallow water.

The performance of the three approaches was compared in Table 3. It is observed that the performance of the convex hull algorithm was high in terms of accurate pinpointing of the true pixel, correctness in the endmember spectral when validated against the field spectra, and terms of the extreme pixel corresponding to each class. This is based on the fact that the spectra obtained from all three algorithms were compared with field spectra, in which CHGS spectra resembled the closest to the field spectra.

Summing up the above-said factors, the PCA technique provides pixels with partially true spectra. The difficulty lies in the delineation of individual, accurate endmembers when they are clustered. PPI on the other hand gives false pixels, which are not spectrally pure for all the land cover components. It is the convex hull approach, which pinpoints accurately the endmember spectra of the land cover component. The CHGS method is geometrically intensive and preferred to be the best [17]. This novel method also is quick to provide the endmembers from the scatterplot with a minimum of two bands. However, the CHGS method demands a high computationally intensive system for the extraction of endmembers with the large number of bands because it considers all the points within the set both interior and exterior points (Alshamrani et al.). This might be because it takes most of the time for the calculation of convex hulls (Heylen and Scheunders).

## 3. Conclusions

This work offers a novel convex hull-based method for the extraction of endmembers. This study has shown that the convex hull's architecture is intended to allow for the proper demarcation of endmembers. This study established that, in comparison to PCA and PPI techniques, the convex hull approach utilizing the Graham scan algorithm offers better endmember spectra. The disadvantages of the PCA technique are in the identification of the extreme pixel on the scatter plot, even if it often offers endmembers of the land cover components. This is due to the clustered distribution of the pixels in the PC scatter plot. On the other hand, the PPI technique results in providing false pixels, representing the land cover components. These disadvantages make the PCA and PPI techniques unviable for subsequent use in spectral unmixing. Convex hull implemented using the Graham scan algorithm delineates the pure pixel and pinpoints the exact number of endmembers. These accurate endmembers would result in accurate proportions of the land cover for better modeling of the terrain. Future research in this field might involve a demonstration of the convex hull theory in the quantification of LULC abundances or fraction pictures of the classes under consideration and utilization of hyperspectral data to derive increased information. The LULC of the given scene would thus be accurately represented by a fraction map as a result.

## Figures and Tables

**Table 1:** DN (8 bit) of endmembers identified using various techniques from study area 1.

Techniques	DN of vegetation			DN of soil			DN of deep water			DN of shallow water		
	b1	b2	b3	b1	b2	b3	b1	b2	b3	b1	b2	b3
PCA	99	64	115	181	164	124	123	85	48	211	175	100
PPI	124	83	123	181	164	124	101	69	66	—	—	—
Convex hull	118	80	120	181	164	124	125	84	49	211	175	100

*Note*. *b*1 = ASTER band 1, *b*2 = ASTER band 2, and *b*3 = ASTER band 3.

**Table 2:** DN (8 bit) of endmembers identified using various techniques from study area 2.

Techniques	DN of vegetation			DN of soil			DN of deep water			DN of shallow water		
	b1	b2	b3	b1	b2	b3	b1	b2	b3	b1	b2	b3
PCA	109	72	102	176	148	126	155	109	52	91	53	46
PPI	104	66	101	170	143	126	155	109	52	93	56	44
Convex hull	111	59	128	177	157	132	162	116	55	93	59	40

*Note*. *b*1 = IRS 1C band 1, *b*2 = IRS 1C band 2, and *b*3 = IRS 1C band 3.

**Table 3:** Performance of the three approaches in the provision of endmembers.

Approaches	Aspects			
	DN	Scatter plot/image	Pinpointing of pure pixels	Correctness of endmember spectra
PCA	Moderately typical	Highly clustered	Poor	Moderate

PPI	Typical	Least number of pure pixels depicted	Moderate	Moderate

Convex hull algorithm	Highly typical	Extreme pixels only depicted	Excellent	Excellent

## Data Availability

The datasets used and/or analyzed during the current study can be obtained from the corresponding author upon reasonable request.
